# Ventricular predominance in biventricular arrhythmogenic cardiomyopathy: Should new subtype criteria be recognized?

**DOI:** 10.1016/j.radcr.2024.03.014

**Published:** 2024-03-28

**Authors:** Santiago Luna-Alcala, Mauricio Garcia-Cardenas, Enrique C. Guerra, Pavel Martinez-Dominguez, Aldo Cabello-Ganem, Leonardo Proaño-Bernal, Cristian A. Chava-Ponte, Arturo Hernandez-Pacherres, Nilda Espinola-Zavaleta

**Affiliations:** a Department of Nuclear Cardiology, National Institute of Cardiology Ignacio Chavez, Mexico City, Mexico; b Department of Magnetic Resonance, San Pablo Clinic, Lima, Peru; c MD–PhD (PECEM) Program, School of Medicine, National Autonomous University of Mexico, Mexico City, Mexico; d Department of Cardiology, Hipolito Unanue National Hospital, Lima, Peru

**Keywords:** Biventricular cardiomyopathy, Left ventricle, Fibrosis, Implantable cardioverter defibrillator, Sudden cardiac death, Ventricular tachycardia

## Abstract

Arrhythmogenic cardiomyopathy is a biventricular disease in which the effect on the left ventricle can be either equivalent to or more severe than that on the right ventricle. It is a rare disease due to its low reported prevalence and typically becomes clinically evident during the second to fourth decade of life. It represents 4% of sudden cardiac death cases referred for autopsy and 10% of cases of unexplained cardiac arrest. We present a challenging case report of a 68-year-old man who arrived at the emergency room with chest discomfort, palpitations, and light-headedness before a syncopal episode with urinary incontinence. During monitoring, ventricular tachycardia was detected and was treated with cardioversion. However, a follow-up electrocardiogram revealed low QRS voltages in limb leads and T-wave inversion in the left precordial leads. The patient underwent a transthoracic echocardiogram and a gadolinium-based magnetic resonance imaging study to evaluate the possibility of acute decompensated heart failure. Both imaging studies revealed low ejection fraction and systolic dysfunction in both right and left ventricles. Furthermore, in the late gadolinium enhancement study, extensive left ventricular subepicardial enhancement with septal predominance in a ring pattern and an irregular morphology of the right ventricular free wall were observed. A diagnosis of biventricular arrhythmogenic cardiomyopathy was established based on the 2020 Padua Criteria. Although there is not a recognized classification within these criteria to establish its subtype, in our case there was a left ventricular predominance due to the presence of additional left ventricular categories.

## Introduction

Arrhythmogenic cardiomyopathy (ACM) is a genetic heart disease which affects the right ventricle (RV), left ventricle (LV), or both. It is considered a rare disease due to its low estimated prevalence in the general population, ranging from 1:2000 to 1:5000 [1]. It typically becomes clinically evident during the second to fourth decade of life but can also rarely appear before puberty or in the elderly [1]. The condition is characterized by fibro-fatty myocardial replacement, which predisposes to lethal scar-related ventricular arrhythmias (VA) and sudden cardiac death (SCD) [2]. Recently, three disease expression patterns have been described by the updated 2020 Padua Criteria: arrhythmogenic RV cardiomyopathy (ARVC), the classic phenotype characterized by RV involvement and absent or minor LV abnormalities, the biventricular phenotype (BVAC) that affects both ventricles, and the left dominant arrhythmogenic cardiomyopathy (LDAC) variant, distinguished by early and predominant LV involvement [2,3]. A multicentric study assessing ventricular involvement patterns using cardiac magnetic resonance reported that BVAC with right predominance was the most frequently observed phenotype, while left predominance, as reported in this case, was the least common [4].

## Case report

**Fig. 1:**
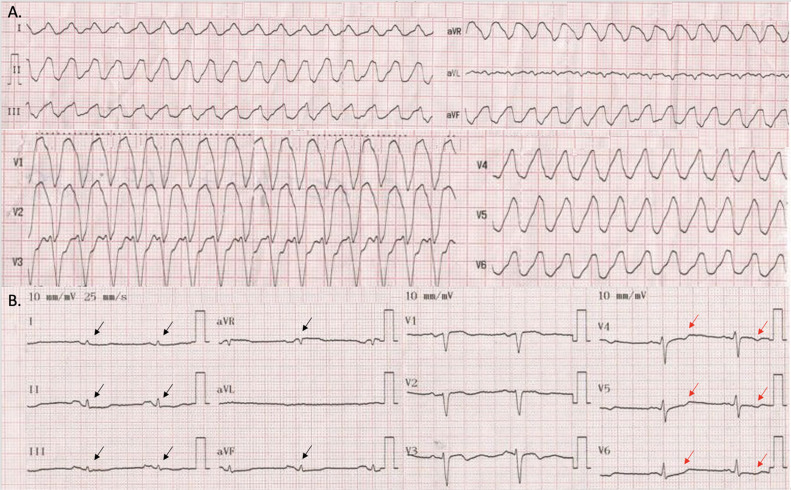
Phenotypic features of arrhythmogenic left ventricular cardiomyopathy. (A) 12-lead ECG revealing a monomorphic ventricular tachycardia. (B) ECG in sinus rhythm after successful pharmacological cardioversion, showing low QRS voltages (<0.5 mV peak to peak) in limb leads (black arrows) and inverted T-waves in the left precordial leads (V4-V6, red arrows).

A 68-year-old man, who was experiencing chest discomfort, palpitations, and light-headedness before a syncopal episode with urinary incontinence, was admitted to the emergency room of a tertiary referral center. He had a medical history of a progressive loss of capacity to perform daily activities, heart failure (HF) and unspecified ventricular tachycardia (VT) treated with electric cardioversion 1 year ago. Upon examination, he was placed on continuous cardiac monitoring. He had a heart rate of 66 bpm, blood pressure of 103/64 mmHg, oxygen saturation of 98% by pulse oximetry, and respiratory rate of 12 breaths/minute. His daily medications included bisoprolol 2.5 mg, spironolactone 25 mg, acetylsalicylic acid 100 mg, and amiodarone 100 mg. His family history was unremarkable, and he denied any cardiovascular symptoms or diseases in first-degree relatives. The initial laboratory work-up showed no abnormalities, except for mild hyponatremia (134 mEq/L). During monitoring, a sustained monomorphic VT with pulse was observed on the 12-lead electrocardiogram (ECG) (Fig. 1A). Therefore, pharmacological cardioversion was started, successfully restoring sinus rhythm. However, the ECG now showed low QRS voltages in limb leads and T-wave inversion in the left precordial leads (V4-V6). (Fig. 1B).

**Fig. 2:**
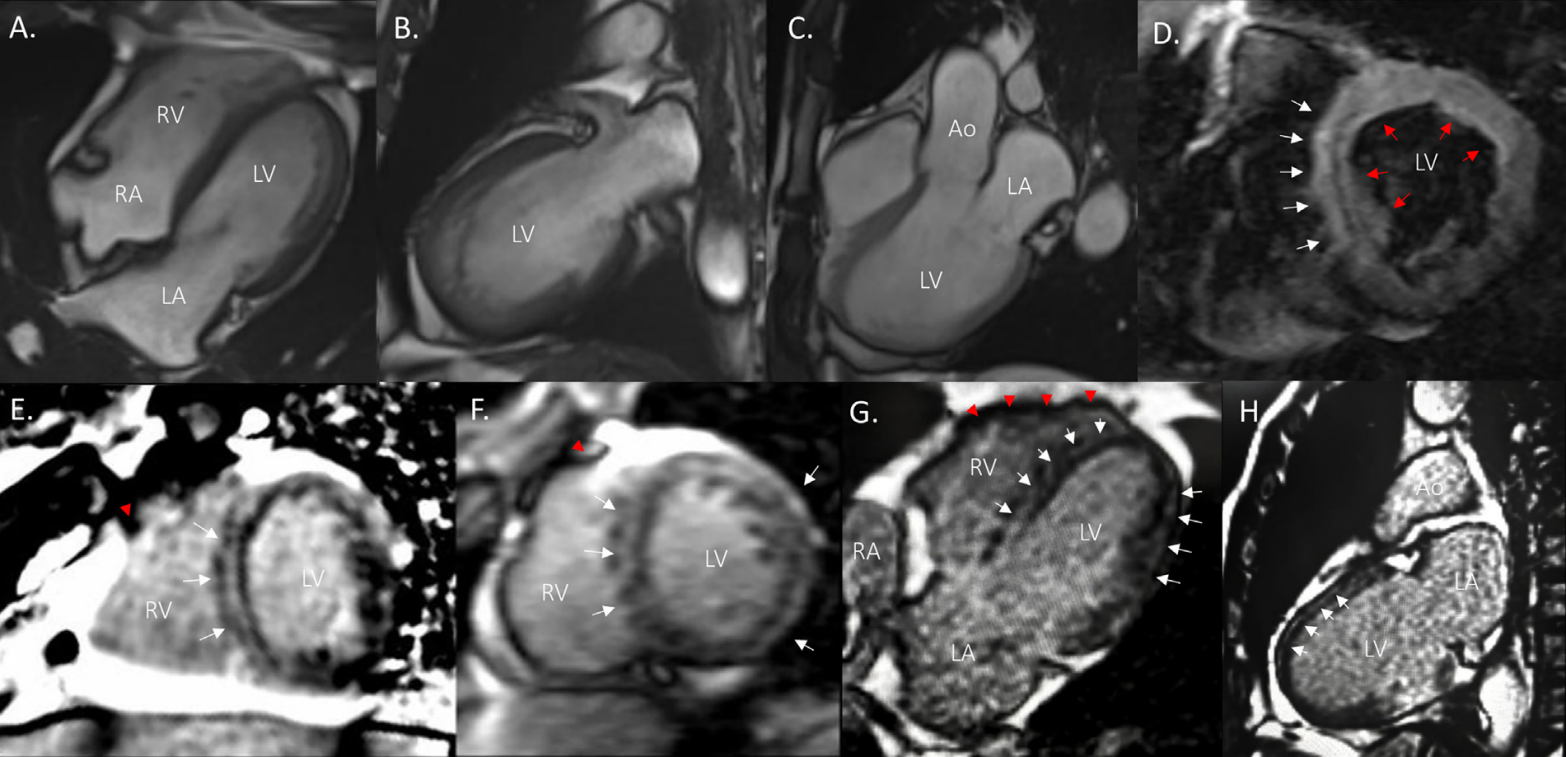
Gadolinium-based magnetic resonance imaging. End-diastolic bSSFP cine (A-C) showing biventricular enlargement. (D) T2-weighted short-axis view showing a linear hypointensity signal in the basal third of the interventricular septum (white arrows) and hyperintensity (red arrows) which likely represents edema. (E, F) LGE short-axis view showing LV ring-pattern subepicardial enhancement (white arrows) and irregular morphology of the RV free wall (red arrowheads). (G) Four-chamber view demonstrating diffuse LGE involving the subepicardial layer of the LV lateral wall and the interventricular septum (white arrows) and showing irregular morphology of the RV free wall (red arrowheads). (H) Vertical long-axis view showing subepicardial LGE at anterior LV wall (white arrows). Ao, Aorta; LA, Left atrium; LV, Left ventricle; RA, Right atrium; RV, Right ventricle.

Due to the suspicion of acute decompensated heart failure, a transthoracic echocardiogram (TTE) was performed, revealing reduced left ventricular (LV) systolic function with a 2D ejection fraction (EF) of 30% (by modified Simpson's method), RV systolic dysfunction (TAPSE of 17 mm; S’ of 5.2 cm/s and RV fractional area change (RVFA) of 27%), enlargement of both atria (left atrial indexed volume of 56.41 mL/m2; right atrial indexed volume of 34.14 mL/m2) and biventricular global hypokinesia. Cardiovascular computed tomography ruled out atherosclerotic plaques (CAD-RADS 0). A gadolinium-based magnetic resonance imaging study was performed with Siemens Magneton Avanto 1.5 Tesla magnet using a rest perfusion protocol for tissue characterization. The long-axis view demonstrated noteworthy enlargement of the left ventricle (end-systolic volume of 95 ml, end-diastolic volume of 144 mL and stroke volume of 49 mL) and the right ventricle (end-systolic volume of 126 mL, end-diastolic volume of 173 mL and stroke volume of 47 mL), moderate LV systolic dysfunction (LVEF 34%) and severe RV systolic dysfunction (RVEF 27%) (Figs. 2A-C). The RV outflow tract did not show any abnormalities with an infundibular portion of 29.5 mm. Pulmonary arteries were confluent with a functional diameter of the pulmonary trunk of 25.5 mm, right pulmonary artery of 19 mm, and left pulmonary artery of 17 mm. The tricuspid valve area was normal (5.6 cm^2^). The LV outflow tract was reported within normal range with an aortic valve area of 3.69 cm^2^. Aortic segments were also within normal range with an aortic annulus of 24 mm, aortic sinuses of 30 mm, sinotubular junction of 27.3 mm, and ascending aorta of 27 mm. A mild mitral insufficiency was reported with distal thickening of the anterior leaflet. The balanced steady-state free precession (bSSFP) cine imaging revealed biventricular enlargement with LV predominance with global hypokinesia and septal dyssynchrony (Supplementary Videos 1,2). T2 weighted imaging short-axis view showed a linear hypointensity in the basal third of the septal wall (Fig. 2D). Late gadolinium enhancement (LGE) in a short-axis view demonstrated extensive LV subepicardial enhancement with septal predominance in a ring pattern and an irregular morphology of the RV free wall (Figs. 2E and F). In addition, a four-chamber view showed diffuse LGE involving the subepicardial layer of the LV free wall and septum, and a vertical long-axis view demonstrated LGE in the anterior LV wall (Figs. 2G and H). These findings led to the diagnosis of BVAC according to the 2020 Padua criteria. However, since he presented additional LV criteria categories, he was considered to have a subtype with LV preferential involvement.

**Fig. 3:**
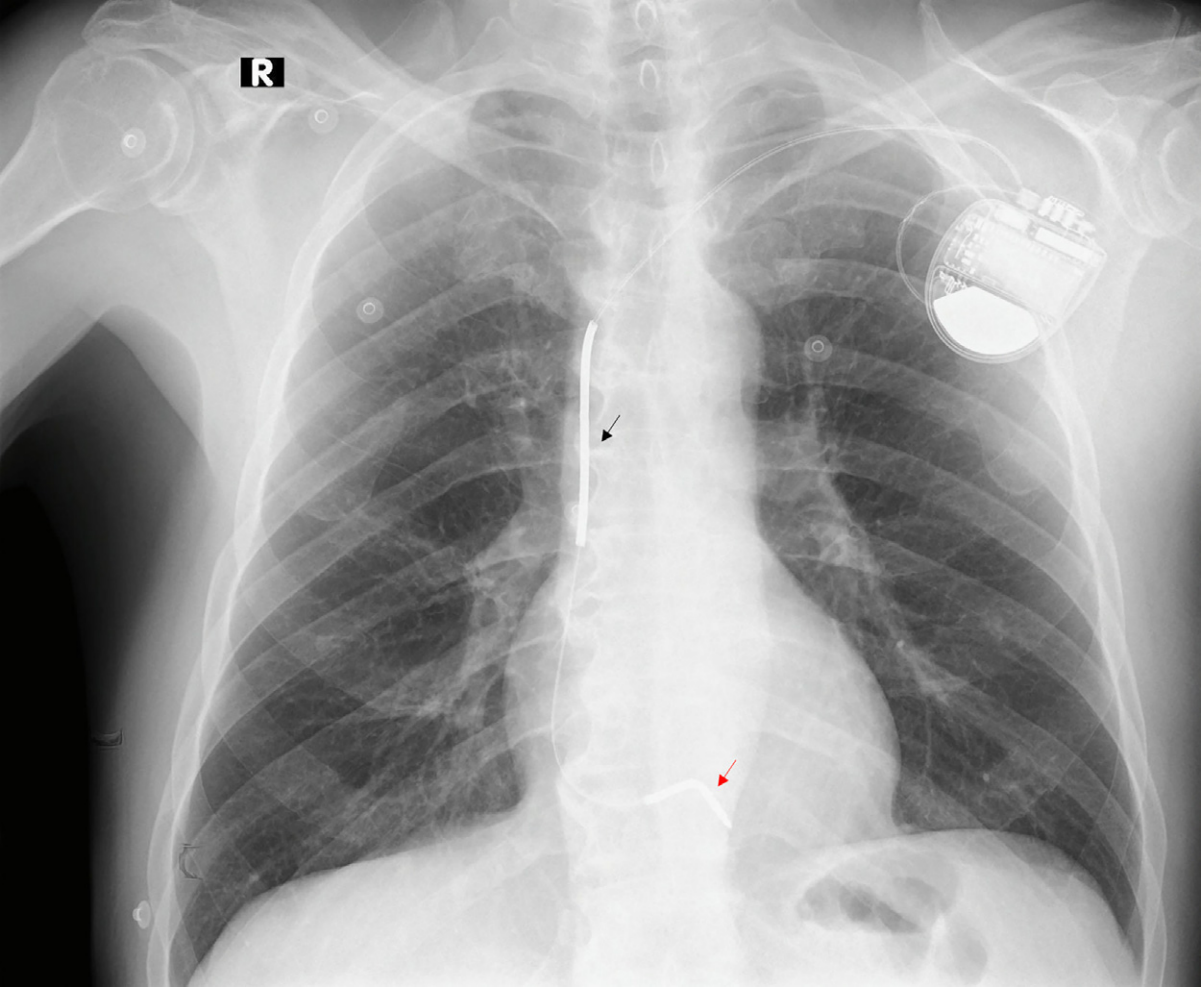
Chest X-ray after ICD placement. Frontal view chest radiography showing a single-chamber pacemaker with its characteristic shock coils in superior vena cava (black arrow) and another that surrounds RV lead (red arrow) in a 68-year-old man with biventricular arrhythmogenic cardiomyopathy with predominant LV involvement.

Days after hospitalization, the patient had a favorable evolution without signs of hemodynamic instability. He was discharged with optimal medical therapy for heart failure and underwent placement of an implantable cardioverter-defibrillator (ICD) as a secondary preventive measure against sudden cardiac death (SCD) (Fig. 3).

## Discussion

The ACM is a congenital heart disease of autosomal dominant inheritance with incomplete penetrance and variable expressivity [5]. It typically manifests between the second and fourth decade of life; early symptoms include palpitations and syncope. However, SCD can sometimes be the first manifestation of the disease, and HF may indicate disease progression [6].

Before the introduction of the 2020 international criteria, commonly referred as the “Padua Criteria”, the 2010 Task Force (TF) guidelines exclusively focused on the features of the RV phenotype [7,8]. These guidelines did not offer diagnostic criteria for left-sided forms and lacked the myocardial tissue characterization findings of gadolinium-enhanced cardiac magnetic resonance (CMR), which is a non-invasive technique for detection of myocardial fibrosis. It only depended on histopathological examination [7]. Therefore, in 2020 an international expert census upgraded the criteria for the diagnosis of ACM and introduced specific diagnostic criteria for the biventricular and left-dominant variants. These include criteria for 6 categories that encompass morpho-functional ventricular abnormalities, structural myocardial tissue alterations, electrocardiographic changes of ventricular depolarization and repolarization, ventricular arrhythmias, and familial and genetic findings [7].

The essential criteria for diagnosis are classified as major, while those that enhance the characterization of the phenotype but are not indispensable are classified as minor [7]. Patients with BVAC phenotype exhibit morpho-functional and/or structural abnormalities in both RV and LV. The diagnostic classification is as follows: “definite” when 2 major criteria, 1 major and 2 minor criteria, or 4 minor criteria from different categories in both ventricles are fulfilled; “borderline” with 1 major and 1 minor, or 3 minor criteria; and “possible” with one major or two minor criteria [7]. According to the Padua Criteria 2020, our patient could be considered as “definite” because the following criteria were met: RV morpho-functional (Minor: Regional RV hypokinesia, and Major: Global RV systolic dysfunction), LV morpho-functional (Minor: Global LV systolic dysfunction with reduced LVEF, and Minor: Regional LV hypokinesia) and structural (Major: LV LGE stria pattern in one bull´s eye segment of the septum) diagnostic anomalies [2]. Although there is not a recognized classification within these criteria to establish the BVAC subtype, as Igual et al. stated, it showed a preferential involvement of the LV based on additional LV criteria categories [4]. Specifically, repolarization (Minor: Inverted T waves in left precordial leads), depolarization (Minor: Low QRS voltages defined as <0.5 mV peak to peak in limb leads), and arrhythmic (Minor: Sustained VT) criteria [2]. We consider that recognizing these BVAC subtypes is essential in planning therapy. In fact, if these phenotypes cannot be diagnosed with the current criteria, appropriate management and prediction of outcomes could be challenging [4].

It is recommended that all patients with suspected or established cardiomyopathy undergo a systematic evaluation to evaluate etiology, assess disease activity, and aid in the detection of extracardiac manifestations. This should include clinical assessment, pedigree analysis (3- to 4-generation family tree), ECG, Holter monitoring, laboratory analysis (e.g., in ARVC, C-reactive protein, liver function, NT-proBNP or BNP, renal function, and troponin), and multimodality imaging [8]. For a comprehensive evaluation of cardiac dimensions and LV and RV systolic as well as LV diastolic function, the use of myocardial deformation imaging (speckle tracking or tissue Doppler) with global longitudinal strain is recommended at initial evaluation (along with CMR) and during follow-up to detect subtle ventricular dysfunction [8].

Desmosomal mutations are the main genetic alterations related to ACM, with *PKP2* gene mutations being the most prevalent and linked to the classic RV phenotype. Additionally, mutations in the *DSG2* and *DSC* genes have been associated with biventricular variants of ACM [9]. Furthermore, genotype-phenotype correlation studies have revealed mutations in non-desmosomal genes, such as *PLN* and *FLNC*, which can lead to left-sided ACM [7,9]. Nonetheless, mutations in non-desmosomal genes have been identified in other cardiomyopathies, including dilated cardiomyopathy (DCM) and neuromuscular cardiomyopathies, resulting in overlapping phenotypes [7]. Therefore, in certain circumstances, the genetic role, in conjunction with the phenotype expression of the disease, is crucial to establish a correct diagnosis.

Since pathogenic or potentially pathogenic variants of desmosomal genes are detected in at least 50% of patients with a high prevalence in young, affected individuals, and considering that ACM is a leading cause of SCD in young athletes under 35 years of age due to ventricular arrhythmias, family genetic testing in patients with a history of ACM in first-degree relatives could be helpful for an early diagnosis [6,10]. This is particularly important for detecting the ALVC presentation, given its worse prognosis compared to isolated RV involvement [7]. Thus, a genetic evaluation of the family enables the early detection of predisposed patients and the proposal of ICD therapy as a primary prevention measure for SCD in young patients at the early stages of the disease when the degree of LV systolic dysfunction is still mild to moderate [7,8].

It is noteworthy that the diagnosis of LDAC without clinical evidence of RV abnormalities requires a documentation of ACM-causing gene mutations, in association with consistent LV structural abnormalities [7]. Therefore, a genetic study was not performed in our patient, as it is not mandatory for BVAC phenotype. This decision was also influenced by the lack of genetic counselors and other barriers that impede family screening and genetic testing in Mexico. Some of these barriers include a low educational level within some population subsets, national geography and infrastructures, low awareness, and minimal knowledge of the disease, and above all the associated costs that genetic studies imply [11].

Diagnosing ACM remains clinically challenging both in the early and later stages due to its mimicry of various pathological conditions. In the early stage, myopericarditis, sarcoidosis, and other inflammatory diseases need to be ruled out, while in the late stage, concerns arise about differentiating ischemic or non-ischemic dilated cardiomyopathies [12].

In patients with suspected ARVC, conditions associated with primary arrhythmias, such as RV outflow tract tachycardia, and Brugada syndrome, must be investigated [2]. Importantly, Brugada syndrome is also associated with VT and SCD and shares characteristics with ACM, such as fibrotic changes, enlargement of the RV outflow tract, and RV focal dyskinesia. However, these patients do not have RV systolic dysfunction or global dilatation [5]. Other diagnoses that should be considered are structural diseases, such as other congenital heart diseases (left to right shunt, Uhl's anomaly and Ebstein's anomaly), pulmonary artery hypertension, and athlete's heart [2]. In last case, intense training in athletes results in physiological cardiac adaptation, including enlargement of the right and left ventricles, ECG anomalies, and arrhythmias, without regional or global systolic dysfunction. Furthermore, it does not include fibrotic changes on magnetic resonance imaging (MRI). In contrast, conditions like ARVC present with global systolic dysfunction, focal dyskinesia, and LGE, which contribute to the diagnostic criteria [5]. RV infarction should also be considered; however, it is associated with a specific vascular territory and lacks the characteristic features of ACM, such as focal hypokinesia and dyskinesia. Furthermore, LGE in RV infarction typically begins at the subendocardial level, in contrast to ARVC, where LGE manifests in the subepicardium or midmyocardium [5].

In the case of LDAC, structural diseases, especially non-ischemic DCM, should be considered. Most DCM patients do not exhibit LGE, but in those who do, it most often occurs in a linear pattern in the mid-wall of the septum [13]. In addition, DCM does not involve the RV, while LDAC is more often associated with malignant ventricular arrhythmias, even at an earlier stage [5]. Moreover, other diagnosis that should be considered are neuromuscular cardiomyopathies (muscular dystrophies and myofibrillar myopathies), myocarditis, sarcoidosis, congenital ventricular aneurysms, and even Chagas’ heart disease [2]. When exploring the possibility of sarcoidosis as a diagnosis, it is essential to consider differential diagnostic criteria, including personal history, such as occupational and/or environmental exposures like those in the lumber industry, rock wool, or glass wool work, and the presence of mediastinal or hilum adenopathy with lung infiltrates at different stages of the disease [5,14,15]. Neither of these criteria was present in our patient. IT is noteworthy that the interventricular septum is affected by noncaseous myocardial necrosis, mural thinning, and focal dyskinesias In comparison to ACM, the interventricular septum is affected by noncaseous myocardial necrosis, mural thinning, and focal dyskinesias [5]. Also, a high-grade atrioventricular block is frequent in these patients, due to atrioventricular node deterioration. Importantly, since isolated cardiac sarcoidosis is a rare entity, the diagnosis should not rely exclusively on MRI or computed tomography [5].

According to the ESC consensus statement for ACM, therapeutic options include lifestyle changes, pharmacological treatment, catheter ablation, an ICD, and, lastly, heart transplantation [8,16].

As regards lifestyle changes, moderate to high-intensity exercise is not recommended for individuals with the ARVC phenotype, including those who are genotype positive/phenotype negative (mainly *PKP2* variant), as it is associated with an acceleration of the disease process [8]. Exercise restriction has been shown to improve clinical outcomes in these patients. Mild to moderate physical activity for up 150 minutes per week is considered safe and is recommended in phenotype-negative individuals [8].

In this case, pharmacological therapy would include a beta-blocker (the first option in ventricular ectopic beats, non-sustained VT, and VT) and the antiarrhythmic drug amiodarone if the arrhythmia is insufficiently managed [8]. The evidence supporting the efficacy of Sotalol remains limited and Flecainide could be considered when a single-agent treatment has failed to control symptoms related to arrhythmias or when autonomic side effects limit the use of beta-blockers [8].

In 2 cases an oral anticoagulant for primary prevention of stroke and thromboembolic events due to ventricular dysfunction was initiated due to a CHA_2_-DS_2_-VASc of 2 [8]. This is also recommended for patients with atrial fibrillation, which has a prevalence of 9%-30% and an annual incidence of 2.1%-2.8% in ARVC patients [8]. Long-term rate control options include beta-blockers, verapamil, or diltiazem, as well as AV node ablation in association with cardiac resynchronization therapy (CRT) [8].

Due to sustained VT, severe ventricular dysfunction, episodes of hemodynamic instability, and the need to prevent SCD, our patient was stratified in the high-risk category. Therefore, an ICD was placed for secondary prevention [8,17]. Catheter ablation of VT is also recommended in patients with incessant VT or frequent appropriate ICD interventions on VT despite maximal pharmacological therapy [8,18]. It has been shown to decrease the incidence of ICD events, VT storm, and mortality by 28% [19]. However, it does not prevent SCD, and it should not be considered as an alternative to ICD implantation in ACM patients with a history of sustained VT [8,18]. Based on the ESC guidelines, catheter ablation should be considered in specialized centers [20]. In our case, it was not done due to lack of resources at the center.

As far as HF management is concerned, the treatment recommendation must be regarded as generic and not specific to the different forms of cardiomyopathy. Medical therapies for HF with reduced ejection fraction (HFrEF) based on randomized controlled trials, including angiotensin-converting enzyme inhibitors, angiotensin receptor neprilysin inhibitors, beta-blockers, mineralocorticoid receptor antagonist, and sodium-glucose co-transporter 2 inhibitors, would be applicable to genetic DCM and other conditions associated with LV dysfunction (e.g. ARVC) [8]. Cardiac resynchronization therapy becomes an available option when medical therapy fails to benefit symptomatic patients with HF, reduced LVEF, and widened QRS complex [21]. Finally, the orthotopic cardiac transplantation is recommended for selected patients with advanced HF or intractable VA refractory to medical, invasive, or device therapy [8]. In our patient, disease progression may ultimately lead to this intervention.

These patients need to be evaluated periodically (every 1-2 years, depending on age, symptoms, and severity) to document new-onset or worsening symptoms, progression of morphological or functional ventricular abnormalities, and episodes of VA to fine-tune the risk of SCD [8]. This approach involves ECG and echocardiography with a multimodality approach for individuals experiencing substantial or unexpected changes in symptoms [8]. However, if the patient requires non-cardiac surgery, peri-operative ECG monitoring is recommended. There should also be a re-evaluation of LV function using echocardiography, along with the measurement of NT-proBNP or BNP levels, especially for those undergoing intermediate or high-risk non-cardiac surgery [8].

In our patient, the pathogenic/likely pathogenic variant was not identified because genetic testing was not available. Consequently, an initial clinical evaluation with ECG and cardiac imaging is recommended for first-degree relatives [8].

## Conclusion

ACM should not be thought exclusively as a RV disease. Other less known variants, such as this case of BVAC with predominant LV involvement, should be on the physician's mind, especially since they encompass specific genetic abnormalities and different clinical characteristics. These BVAC subtypes should be recognized by international committees and criteria categories to diagnose and establish recommendations for their management and potential predicted outcomes developed. These conditions should be suspected in patients of all ages with VA of unknown origin, non-obstructed coronary images, and DCM with associated arrhythmias. Catheter ablation can be considered as an adjunct to ICD therapy which is the main medical intervention to prevent SCD, the most life-threatening consequence.

## Case report limitation

The main limitation of this report was the inability to conduct genetic testing on the patient and his family for molecular phenotype characterization. While it is not a mandatory criterion for diagnosis, such testing could contribute to the management, prognosis, and implementation of primary preventive measures for first-degree descendants.

## Future research direction

ACM poses significant challenges in terms of research and clinical management. First and foremost, there is a notable absence of randomized controlled trials evaluating therapies for the effective management of arrhythmias and heart failure associated with this disease. Moreover, the available studies examining the impact of exercise on ACM remain predominantly retrospective, highlighting the need for more rigorous investigations in this area. Furthermore, our understanding of the incidence and prognosis of heart failure in ACM patients is limited, warranting more comprehensive studies to enhance our knowledge. In addition, predominant biventricular ACM subtypes need to be recognized to better characterize and manage them, stratify their risks, and determine prognosis for both the patients and their descendants. Lastly, the frequency and optimal mode of clinical screening for asymptomatic family members with a predisposition to ACM lack sufficient exploration. Addressing these research gaps will be crucial for advancing our understanding and developing evidence-based strategies for the diagnosis and management of ACM in affected individuals and their at-risk family members.

## Patient consent

The authors confirm that written patient consent for publication has been obtained, in line with the COPE best practice guidelines, and that the individuals being reported on are aware of the possible consequences of the reporting. This study complies with the Declaration of Helsinki, and it was approved by the local ethics committee.

## Appendix Supplementary materials

**Supplemental Video 1: [Cardiac magnetic resonance imaging.** Four, two and three chamber views bSSFP cine images revealed biventricular enlargement with LV predominance with global hypokinesia and septal dyssynchrony.] Image, video 1

**Supplemental Video 2: [Cardiac magnetic resonance imaging.** Short axis bSSFP cine images revealed global biventricular hypokinesia with septal dyssynchrony.] Image, video 2

## References

[1] Pilichou K, Thiene G, Bauce B, Rigato I, Lazzarini E, Migliore F (2016). Arrhythmogenic cardiomyopathy. Orphanet J Rare Dis.

[2] Corrado D, Perazzolo Marra M, Zorzi A, Beffagna G, Cipriani A, Lazzari MD (2020). Diagnosis of arrhythmogenic cardiomyopathy: the Padua criteria. Int J Cardiol.

[3] Sen-Chowdhry S, Syrris P, Prasad SK, Hughes SE, Merrified R, Ward D (2008). Left-dominant arrhythmogenic cardiomyopathy. J Am Coll Cardiol.

[4] Igual B, Zorio E, Maceira A, Estornell J, Lopez-Lereu MP, Monmeneu JV (2011). Arrhythmogenic cardiomyopathy. patterns of ventricular involvement using cardiac magnetic resonance. Revista Española de Cardiología (English Edition).

[5] Manole S, Pintican R, Popa G, Rancea R, Dadarlat-Pop A, Vulturar R (2022). Diagnostic challenges in rare causes of arrhythmogenic cardiomyopathy—the role of cardiac MRI. JPM.

[6] Cicenia M, Drago F. (2022). Arrhythmogenic cardiomyopathy: diagnosis, evolution, risk stratification and pediatric population—where are we?. JCDD.

[7] Corrado D, Basso C. (2022). Arrhythmogenic left ventricular cardiomyopathy. Heart.

[8] Arbelo E, Protonotarios A, Gimeno JR, Arbustini E, Barriales-Villa R, Basso C (2023). 2023 ESC guidelines for the management of cardiomyopathies. Eur Heart J.

[9] Mattesi G, Cipriani A, Bauce B, Rigato I, Zorzi A, Corrado D. (2021). Arrhythmogenic left ventricular cardiomyopathy: genotype-phenotype correlations and new diagnostic criteria. J Clin Med.

[10] Akdis D (2016). Department of Cardiology, University Heart Center, Zurich, Switzerland, Brunckhorst C, Department of Cardiology, University Heart Center, Zurich, Switzerland, Duru F, Department of Cardiology, University Heart Center, Zurich, Switzerland, et al. Arrhythmogenic Cardiomyopathy: Electrical and Structural Phenotypes. Arrhythmia Electrophysiol Rev.

[11] Germain DP, Moiseev S, Suárez-Obando F, Al Ismaili F, Al Khawaja H, Altarescu G (2021). The benefits and challenges of family genetic testing in rare genetic diseases—lessons from Fabry disease. Molec Gen Gen Med.

[12] Spadotto A, Morabito D, Carecci A, Massaro G, Statuto G, Angeletti A (2022). The challenges of diagnosis and treatment of arrhythmogenic cardiomyopathy: are we there yet?. Rev Cardiovasc Med.

[13] Halliday BP, Baksi AJ, Gulati A, Ali A, Newsome S, Izgi C (2019). Outcome in dilated cardiomyopathy related to the extent, location, and pattern of late gadolinium enhancement. JACC: Cardiovasc Imaging.

[14] Liu H, Patel D, Welch AM, Wilson C, Mroz MM, Li L (2016). Association between occupational exposures and sarcoidosis. Chest.

[15] Vereckei A, Besenyi Z, Nagy V, Radics B, Vágó H, Jenei Z (2024). Cardiac sarcoidosis: a comprehensive clinical review. Rev Cardiovasc Med.

[16] Corrado D, Wichter T, Link MS, Hauer R, Marchlinski F, Anastasakis A (2015). Treatment of arrhythmogenic right ventricular cardiomyopathy/dysplasia: an international task force consensus statement. Eur Heart J.

[17] Gasperetti A, James CA, Carrick RT, Protonotarios A, Te Riele ASJM, Cadrin-Tourigny J (2023). Arrhythmic risk stratification in arrhythmogenic right ventricular cardiomyopathy. Europace.

[18] Migliore F, Mattesi G, Zorzi A, Bauce B, Rigato I, Corrado D (2021). Arrhythmogenic cardiomyopathy—current treatment and future options. JCM.

[19] Samuel M, Healey JS, Nault I, Sterns LD, Essebag V, Gray C (2023). Ventricular tachycardia and ICD therapy burden with catheter ablation versus escalated antiarrhythmic drug therapy. JACC: Clin Electrophysiol.

[20] Zeppenfeld K, Tfelt-Hansen J, De Riva M, Winkel BG, Behr ER, Blom NA (2022). 2022 ESC Guidelines for the management of patients with ventricular arrhythmias and the prevention of sudden cardiac death. Eur Heart J.

[21] Maniar Y, Gilotra NA, Scheel PJ. (2023). Management strategies in arrhythmogenic cardiomyopathy across the spectrum of ventricular involvement. Biomedicines.

